# Exploring Collaboration to Center the Deaf Community in Sign Language AI

**DOI:** 10.1145/3663547.3746390

**Published:** 2025

**Authors:** Rie Kamikubo, Abraham Glasser, Alex X Lu, Hal Daumé, Hernisa Kacorri, Danielle Bragg

**Affiliations:** University of Maryland, College Park, Maryland, USA; Gallaudet University, Washington, District of Columbia, USA; Microsoft Research, Cambridge, Massachusetts, USA; University of Maryland, College Park, Maryland, USA; University of Maryland, College Park, Maryland, USA; Microsoft Research, Cambridge, Massachusetts, USA

**Keywords:** Deaf and Hard of Hearing, Machine Learning, Sign Language Technology, Interdisciplinary Collaboration

## Abstract

Sign language processing holds great promise for advancing societal inclusivity, yet it often excludes meaningful participation from the Deaf community, raising ethical and practical concerns about the applicability of AI solutions to their needs. This paper addresses these gaps through two interrelated studies. First, surveys identify differences in priorities and expectations between machine learning (ML) practitioners and Deaf American Sign Language (ASL) signers. Second, paired co-design sessions bring ML and ASL experts together to generate guiding questions that support practices for aligning AI development with community goals. Our findings reveal critical points of friction that reflect deeper systemic and epistemic barriers to effective collaboration. By synthesizing unique and shared insights from both groups, we provide empirically grounded resources to guide collaborative frameworks that promote the agency and expertise of the Deaf community. This research paves actionable pathways toward equitable, community-centered advancements in AI.

## 1 Introduction

The development of technologies for sign languages, such as American Sign Language (ASL), has attracted attention in machine learning (ML) and artificial intelligence (AI)—*e.g.*, computer vision for sign language recognition [5, 64, 67] and natural language processing for sign language translation [47, 57, 87]. Progress in these areas, however, requires interdisciplinary expertise, including sign language linguistics and understanding of Deaf culture. Limited experience in these domains increases the risk of creating tools that are ineffective, culturally insensitive, or harmful to the Deaf community [27, 38, 75]. Yet, AI research for sign language remains siloed and often lacks critical collaboration with Deaf people [13], perpetuating fairness issues and hindering the development of inclusive, equitable solutions [12, 34].

In this paper, we respond to the interdisciplinary and participatory call for action in sign language processing, a field of AI where ML methods are often applied and advanced for sign language [13]. We explore the challenges and opportunities of fostering collaboration by integrating stakeholders’ diverse expertise in the project lifecycle. Our work focuses on Deaf or Hard of Hearing (DHH) signers, specifically those who use **ASL** (referred to as ASL signers in this paper), and **ML** practitioners as key stakeholders in establishing an interdisciplinary foundation, combining the technical expertise of ML with the lived experiences and domain knowledge of sign language. We aim to support these collaborative practices, recognizing that practice tends to be far from the ideal [19, 20]. While frameworks and tools exist to guide sign language technology development [2, 63], there remains a lack of holistic resources that address broader collaboration needs, with existing resources often limited to specific tasks, such as data collection [1, 23, 68, 85]. Even when collaboration appears to be in place, systemic issues of power imbalances and tokenism continue to surface [20, 24].

This research aims to inform collaboration for more inclusive and responsible development of sign language technologies. We engage key stakeholders through two studies. First, through a pair of surveys, we uncover potential points of friction and misalignment among ASL signers (N=10) and ML practitioners (N=35), capturing their motivations, concerns, and expectations for sign language AI projects. Second, we conduct a paired co-design study that brings together Deaf ASL experts (N=8) and ML experts (N=8)—individuals with specific qualifications and experience—to collaborate in the early stages of hypothetical sign language AI projects. Using a guided inquiry process [7], participants in these sessions generate and explore questions to guide discussions on problem formulation and project requirements.

Our findings reveal barriers and opportunities for collaboration between Deaf and ML community members. From the surveys, we identify barriers such as differing incentives, priorities, and epistemic challenges *e.g.* limited scope of inquiry in problem formulation for ML practitioners. For ASL signers, obstacles included communication inaccessibility and a lack of Deaf awareness among hearing collaborators. Indeed, our surveys show that misconceptions persist even among those with sign language processing experience. From the co-design sessions, we find that collaboration-driving questions often center on shared themes, with ‘objectives’, ‘methodology’, ‘timeline’, and ‘stakeholder analysis’ emerging as the most prominent. Yet even within shared themes and in seemingly similar questions, ML and ASL experts take different approaches, reflecting a tension between task-oriented inquiry and community-centered reflection. By sharing these questions, we hope to point the research community to opportunities to bridge these gaps, such as scoping projects that integrate technical and societal considerations, fostering discussions on community engagement and the impact of these technologies, and weighing benefits and risks.

This research provides actionable insights for fostering interdisciplinary efforts in AI for sign language. Based on our findings, we outline pathways for inclusive, community-driven initiatives that empower Deaf people and promote ethical and responsible technology development. Our contributions are both empirical and methodological. Our surveys reveal the current state of technology practices, identifying areas for improvement to initiate collaboration between ML practitioners and Deaf signers. More so, the themes that emerged from the guiding questions in our co-design study inform a framework for facilitating stakeholder discussions and addressing misalignments. These resources help bridge gaps between communities, deepen understanding of their unique and shared perspectives, and support practitioners in moving beyond siloed practices toward greater involvement in AI innovation.

## 2 Related Work

As AI research expands to create innovative and scalable solutions for a more inclusive society, calls for diverse representation in defining problems and goals for AI-infused systems are growing [25, 28, 54, 77]. Similar discussions are occurring in accessibility [12, 56, 70, 81], aiming to facilitate equitable and empowering technology design, development, and deployment. In this section, we cover prior literature on involving diverse stakeholders, particularly the Deaf community, and situate these discussions in the context of sign language technology.

### 2.1 Participation in AI and Accessibility

A growing body of work emphasizes participatory approaches in AI [4, 9, 22, 88], highlighting their importance in understanding and representing the needs, desires, and perspectives of historically marginalized communities [9]. Approaches such as participatory data collection [41, 45], respectful participation frameworks [9, 44], and motivational incentives [74] facilitate active and meaningful inclusion in shaping the future of AI-infused technologies.

While there is demand for increasing representation of disabled people in shaping design problems and solutions, effective participation is hindered by ethical and methodological challenges in technology practices [6, 76]. For example, prior research highlights issues such as the limited accessibility of design tools for mixed-ability teams [46, 70, 71]. Societal and cultural norms further impede meaningful involvement of the disability community. Power dynamics within teams have been widely discussed in accessibility research, highlighting deeper systemic issues that undermine agency and ownership [73, 76, 86]. These challenges are particularly pronounced in research involving the Deaf community, where distrust toward hearing researchers, particularly those lacking sign language proficiency, exacerbates these issues [73]. While interpreters can mediate communication between signing Deaf and hearing team members, unequal participation can still manifest as lag in turn-taking or delays in engagement [37]. More so, disparities at the root, such as limited access to critical resources in STEM education [65], can lead to exclusionary processes and outcomes (*e.g.*, the absence of Deaf leadership [24]), further reinforcing structural imbalances in collaboration [36].

To overcome barriers to participation, researchers in human-computer interaction (HCI) and accessibility have pursued more inclusive approaches to technology design, such as developing resources to bridge gaps in cross-cultural or disciplinary contexts [26, 59] and adopting accessible co-design methodologies [3, 69]. These efforts can foster collaboration by integrating the lived experiences and perspectives of disabled people into the development process [16, 32]. Our work builds on these discussions in the context of sign language technology research. We aim to deepen our understanding of the barriers to effective collaboration with the Deaf community and inform the development of necessary supports to address the question: *“How can people work together effectively in this space?”* [12].

### 2.2 Sign Language Technology Development

Given the societal and structural barriers limiting the Deaf community’s participation and leadership, collaboration is a pragmatic and urgent necessity for advancing sign language technology. While empowering Deaf people to lead these efforts remains a vital long-term goal, the growing interest in developing methods for sign language technology within the broader AI field [42, 84] underscores the immediate need to engage the Deaf community in this interdisciplinary space. Sign language-specific machine learning tasks present unique obstacles, requiring a nuanced understanding of linguistic and cultural contexts. For example, translating sign language to spoken language involves a visual recognition task to detect and label signs as glosses^1^, followed by a natural language processing task of translating between the gloss and spoken language text. However, glosses are not simple transcripts of the corresponding spoken language [62]. The unique grammar and linguistic properties limit the applicability of methods established for spoken or written languages (*e.g.*, machine translation) [13]. In addition, the lack of annotated datasets complicates modeling, as annotations require expertise in sign language linguistics and considerations of social and regional variations [21, 58].

Despite the interdisciplinary nature of sign language technology development, research in this field often occurs within isolated silos [13]. These practices mirror broader patterns of marginalization, where Deaf people are often not centered in the process of research and design [83]. This exclusion underscores systemic resource issues, including the lack of accessible training pipelines for Deaf people in AI and related fields [24]. Hence, it is not surprising that misalignments between the needs of the Deaf community and the implications of technology use are commonly observed, with proposed solutions viewed as “disability dongles”^2^ [27, 38]. Without an understanding of Deaf culture and lived experiences, practitioners risk misrepresenting problems, leading to adverse effects in real-world deployment [12].

Frameworks and tools have been proposed to guide the development of sign language technologies that address the needs of the Deaf community [2, 63]. However, many focus narrowly on specific tasks, such as data collection [1, 12, 23, 68], lacking clear guidance on initiating and sustaining meaningful collaboration with the Deaf community throughout the development. While Deaf professionals are already contributing to this field, concerns persist about downstream challenges when practitioners, such as those in machine learning without lived experience of the Deaf community, lead these efforts [83]. Our work aims for a more holistic approach, addressing the need for tools and strategies to foster collaboration and empower the Deaf community in shaping technologies that directly impact their lives.

## 3 Methods

We designed a two-part study to understand potential collaboration barriers and explore opportunities for bridging gaps in interdisciplinary and cross-cultural teams working on sign language AI. Our study involved: 1) surveys capturing perspectives from both ASL signers and ML practitioners to identify misaligned expectations (Sec. 3.1), and 2) paired co-design sessions with ASL and ML experts^3^ to explore resources and strategies for addressing these misalignments (Sec. 3.2). Ethics approval was obtained through institutional review boards at the study team’s institutions. The team includes both Deaf and hearing researchers from U.S.-based academic and industry institutions.

### 3.1 Study 1: Surveys

We designed two surveys targeting ML practitioners and ASL signers. The surveys included overlapping questions, summarized in Table 1, to compare perspectives and identify points of alignment or friction. In total, 26 questions were presented, with 12 shared between both groups: 35 ML practitioners (19 with and 16 without sign language processing experience) and 10 ASL signers (demographics in Appendix A). For every survey submission, US$10 was donated to a Deaf advocacy organization.

#### 3.1.1 Recruitment and Participants.

We focused on ASL signers within the U.S., considering the diversity of values across different sign language communities; however, we acknowledge the linguistic and cultural variations that exist within a single community [58]. In contrast, we made a broader call for ML practitioners, including those with and without prior experience in sign language processing, to better understand the current landscape. This reflects the growing interest within the field of AI [42, 84], where machine learning methods are often applied and advanced for sign language without a proper understanding of the linguistic and cultural contexts [13]. We anticipated those without experience to show less alignment with Deaf community priorities. The experienced group was intended to serve as an “upper baseline” to examine whether prior exposure would correlate with higher Deaf awareness and thus reduce that gap. Notably, as shown Section 4, we find that misconceptions persist even among ML practitioners with prior experience in sign language processing. In our statistical analysis, conducted only between the two ML subgroups on Deaf awareness items, we did not observe a significant difference. Therefore, we present the two ML subgroups jointly in this study.

We circulated the study call with relevant survey links to the intended groups, using tailored outreach strategies for each:

##### ASL Signers.

We invited DHH ASL signers—those with lived experience of sign language and cultural knowledge. While individual factors like geographic region, age, gender, and education could add variety, we did not sample by these for practical reasons. Recruitment is already a challenge when working with smaller populations. Recruitment was done through Deaf community listservs, social media, and snowball sampling. We relied on personal contacts, including Deaf professionals and ASL teachers working with Deaf communities, to circulate the survey within their networks.

##### ML Practitioners.

We invited practitioners with machine learning experience across diverse domains (*e.g.*, computer vision, NLP, deep learning) and fields (*e.g.*, HCI, accessibility, linguistics), regardless of their sign language knowledge or experience in sign language processing. Using convenience sampling, we recruited participants via subscribed research mailing lists (*e.g.*, ACL web, connectionists), topical mailing lists (*e.g.*, ML or HCI), social media, and targeted outreach to authors of relevant publications.

#### 3.1.2 Survey Design.

The surveys covered the following themes:

##### Background.

Both surveys included closed-ended demographic questions, such as age, gender, education, occupation, primary language, and DHH identity. Participants also self-rated their ASL proficiency using the ASL Language Proficiency Interview (ASLPI) scale [80] from Level 0–5. ML practitioners were asked about their experience in ML, involvement in sign language projects, and awareness of Deaf culture, including appropriate terminology and identity. ASL signers responded to questions about their experiences in cross-cultural and cross-disciplinary collaboration, particularly with hearing people, and their awareness and exposure to ML.

##### Motivations/Challenges.

To uncover misaligned priorities and needs in pursuit of collaboration, we tailored survey questions to the motivations and challenges of ML practitioners and ASL signers. ML practitioners were asked about their experiences in ML and/or sign language processing, including factors influencing problem choice. ASL signers were asked about factors encouraging collaboration with ML experts, along with pain points and accommodation needs. Most questions were open-ended, with a few closed-ended questions serving as branching points.

##### Expectations.

ML practitioners and ASL signers received the same set of closed-ended questions about preferences for problem selection (*e.g.*, recognition vs. translation), evaluation criteria (*e.g.*, accuracy vs. speed), timelines for technological advancements, and task priorities when working with sign language data and models.

### 3.2 Study 2: Paired Co-design

To inform interdisciplinary and cross-cultural collaboration with the Deaf community, we conducted 8 paired co-design sessions via Zoom, each pairing an ASL expert with an ML expert to collaborate on a hypothetical sign language AI project. Pairing instead of groups allowed each participant to share input directly with their partner.

The pairs went through a guided inquiry process [7] to generate questions they considered important to drive collaboration discussions. We analyzed these questions to understand their priorities in forming interdisciplinary and cross-cultural AI teams and the value of each individual/community to address potential misalignment.

#### 3.2.1 Recruitment and Participants.

Given the challenges in recruiting participants with specific expertise and experiences, we employed non-probability sampling methods such as snowball sampling and word of mouth that can help with the recruitment. Participants were pre-screened to ensure expertise in ASL or ML. Demographics are provided in Appendix B.

##### ASL Experts

To meet the eligibility criteria of this group, participants are proficient in ASL, identify as DHH, and have been exposed to technology-related projects. We prioritized ASL experts who had previously participated in or worked on projects related to technology to gain insights into interdisciplinary collaboration in this space. We recruited through listservs associated with Deaf community networks, and through snowball recruiting through relevant contacts including Deaf professionals and accessibility researchers working with Deaf communities.

##### ML Experts

To meet the eligibility criteria of this group, participants are proficient in machine learning and have been exposed to sign language, Deaf culture and/or sign language-related projects. We prioritized ML experts who are knowledgeable about stakeholders and users in this space to enable informed discussions surrounding collaboration challenges and opportunities. We recruited ML experts via ML- and research-related email lists, and through snowball sampling with practitioners specifically working in sign language processing, such as authors who published relevant work in academic venues (*e.g.*, ACL Anthology, LREC, CVF), organizers of relevant workshops (*e.g.*, WMT-SLT), and sign language dataset creators (populated from IncluSet [43]).

#### 3.2.2 Session Design.

Each co-design session took 2 hours, with the last 30 minutes spent on a post-session questionnaire. At completion, participants received a US$50 digital gift card as compensation. A Deaf and hearing researcher facilitated each session, and interpreters were provided on the call. All Zoom sessions were recorded, with consent, for further analysis.

##### Question generation.

**Figure 1:**
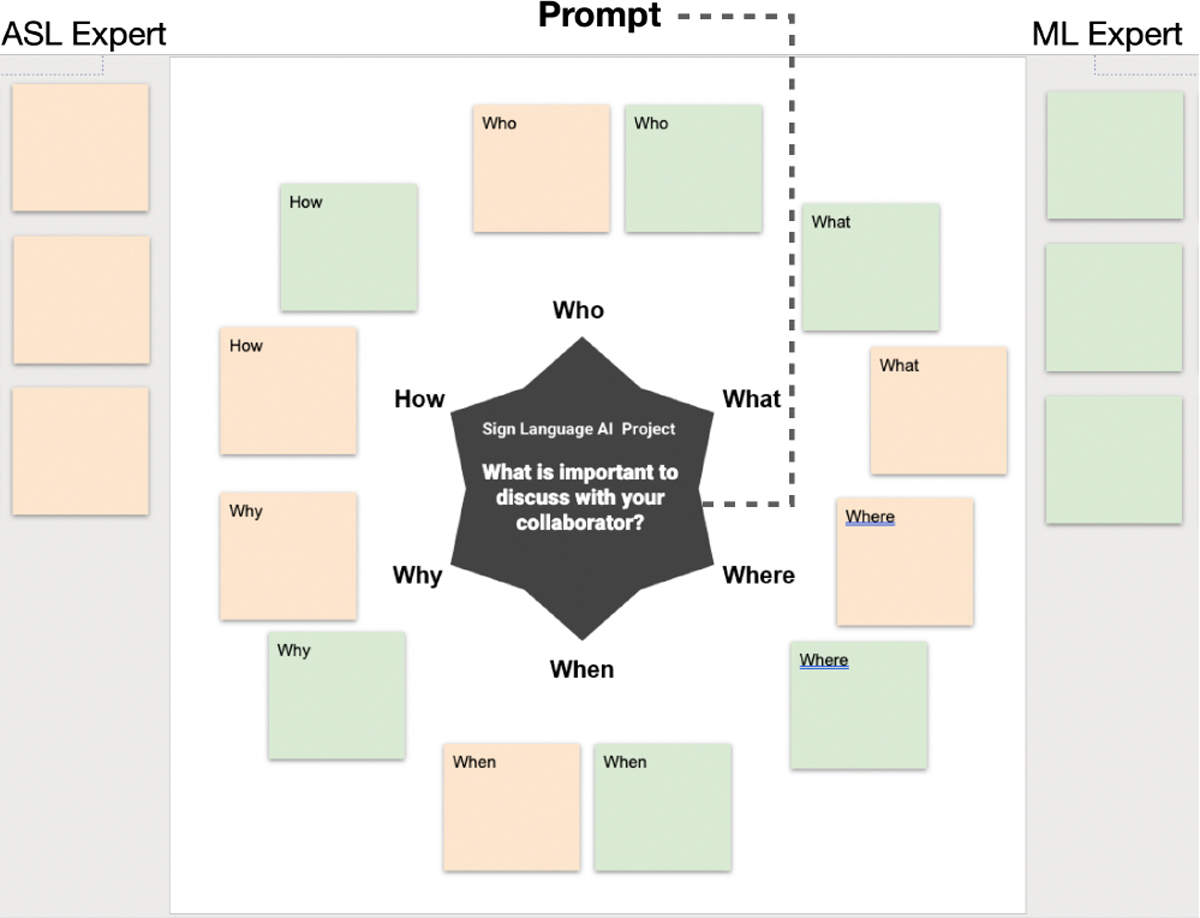
Participants write down questions in “sticky notes” – colored text boxes – provided at the side of the slide. The questions follow the format of Who, What, Where, When, Why, and How in response to the given prompt.

Leveraging guided inquiry, to facilitate discussions we asked each pair of participants to engage in an activity called *starbursting* [7], a form of brainstorming that focuses on generating questions rather than answers. During the co-design session, they received a unique link to a browser-based slide deck to write down questions on virtual sticky notes (Fig. 1). Participants were asked to imagine that they were starting a collaboration on a sign language AI project, and generated questions in response to a prompt: “What is important to discuss with your collaborator?” Participants were asked to write down questions that came to mind to structure points of discussion on project requirements, such as the topic, problem, task, or roles. The starbursting activity used written English, which could pose language barriers. To address this, ASL interpreters facilitated communication during the session, alongside one Deaf, native ASL signer on the research team and one hearing researcher. Additionally, as shown in Appendix B, our recruitment of highly informed, professionalized DHH ASL signers, all holding a Bachelor’s degree or higher, meant participants were more likely to be strongly bilingual.

##### Question update.

After each prompt, pairs discussed the generated questions. Each participant could vote for two questions they deemed most important, one of their own and one of their collaborator’s, with facilitators also voting. The facilitators used the votes to guide the discussion. After discussing, pairs had the opportunity to add new questions or comments to those not previously covered, with prompts for clarifications, revisions, or removals.

##### Questionnaire.

Lastly, participants completed a closing questionnaire containing a mixture of open-ended and closed-ended questions. We asked about their demographics, experience with collaboration on technology or sign language projects, and attitudes toward potential collaboration.

### 3.3 Analysis

#### 3.3.1 Surveys.

The collected data consisted of both quantitative responses and shorter qualitative responses from a mixture of open-ended and close-ended survey questions. For quantitative data, simple descriptive statistics were used, including average ratings, average rank scores, and the percentage of responses for categorical questions for both ML practitioners and ASL signers. We also performed a statistical analysis to compare the two ML subgroups. Specifically, we explored differences between ML practitioners with and without experience in sign language processing, based on their correct response rates to a series of true/false questions on Deaf awareness. Given the nature of the data, we applied the Mann-Whitney U test, a non-parametric method, to compare the distributions between the two independent subgroups.

For qualitative data, we used a content analysis approach to identify patterns in responses [49]. Responses were categorized based on similarities and differences, and the frequency of similar responses was counted to better understand the phenomenon. This process helped synthesize the findings, including barriers faced by ML practitioners in sign language processing and by ASL signers in cross-cultural and cross-disciplinary collaboration. No statistical tests were applied between the ASL signer group and ML practitioners both due to size and qualitative focus.

#### 3.3.2 Co-design.

All co-design sessions were transcribed, and we retained video recordings to reference both the ASL expert’s signing and the interpreter’s voicing. This step was critical, as meaning can often shift or be lost in translation. For example, participant ASL-P3 intended to ask, *‘What should a project focus on – the people, the functionality of the technology, or how it’s presented to the community?’* but the question was written as, *‘Is it the people, function, or exposure?’* We analyzed the guiding questions generated from 16 participants and questionnaire responses to contextualize our findings. Participants contributed 150 questions that reflected their exploration of key project considerations. To understand participants’ unique and shared perspectives, one researcher initially grouped questions using a digital whiteboard (Miro), and data analysis meetings involving Deaf and hearing researchers were subsequently held to refine these groupings. The digital whiteboard allowed researchers to collaboratively add comments, adjust groupings, and propose thematic labels. We used the transcripts and videos to better understand the questions and help our categories reflect what participants meant.

## 4 Survey Findings

In this section, we report perspectives and trends observed among ML practitioners (N=35) and ASL signers (N=10). Appendix A provides detailed participant demographics.

### 4.1 Participant Motivations and Concerns

**Figure 2:**
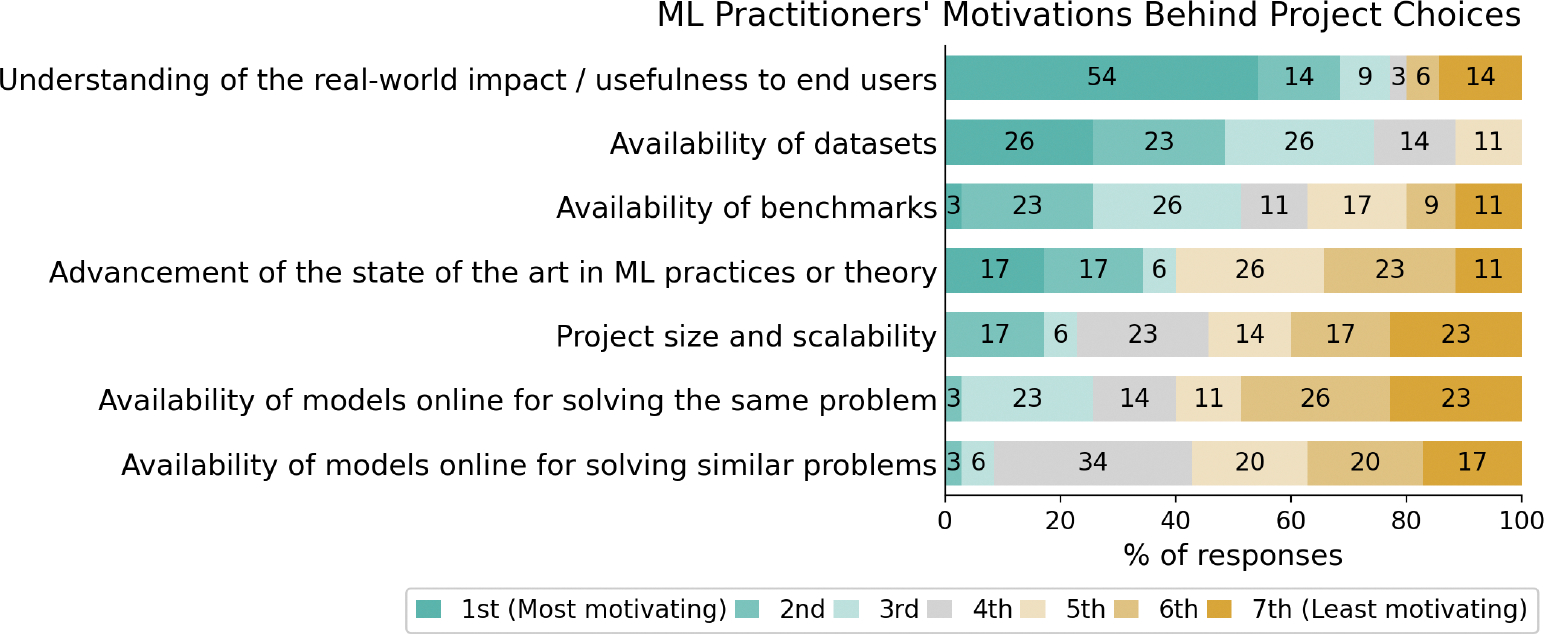
A breakdown of responses from ML practitioners, ranking the motivating factors behind project choices in ML.

Regarding **ML practitioners’ motivations** behind their project choices, the majority (77%) ranked real-world impact and usefulness to end users within their top 3 motivating factors (Fig. 2). Availability of datasets and benchmarks also ranked highly.

We further explore motivations to work on sign language projects, based on the perspectives of the 19 ML practitioners who reported having experience in sign language processing. These participants highlighted various external sources of motivation, including societal impact (21%), professional circumstances (26%), and funding (16%). One ML practitioner shared, *“It was the option that I thought would have more societal impact from the ones that my advisor provided. And although I had never used sign language I was very curious about it.”* Personal factors, such as being a member of the Deaf community (16%), also played a role. Additionally, many (42%) expressed a desire to apply or advance ML methods to work in this space, as stated by an ML practitioner: *“Signed languages should be part of NLP. Sign language processing problems are interesting and challenging... a lot of progress can be achieved.”*

Next, we explored **ML practitioners’ concerns** specific to sign language processing. Among ML practitioners with relevant experience, a majority (58%) highlighted the lack of data and annotation as a significant challenge. This problem was often reported alongside issues such as limited methodological tools (26%) and computing resources (21%). Domain complexity was another reported challenge (26%), partly due to *“not having a native user level of sign language knowledge”* and the computation difficulty to capture grammatical nuances. A few ML practitioners (11%) voiced concerns about facing social resistance from the Deaf community as hearing researchers.

**Figure 3:**
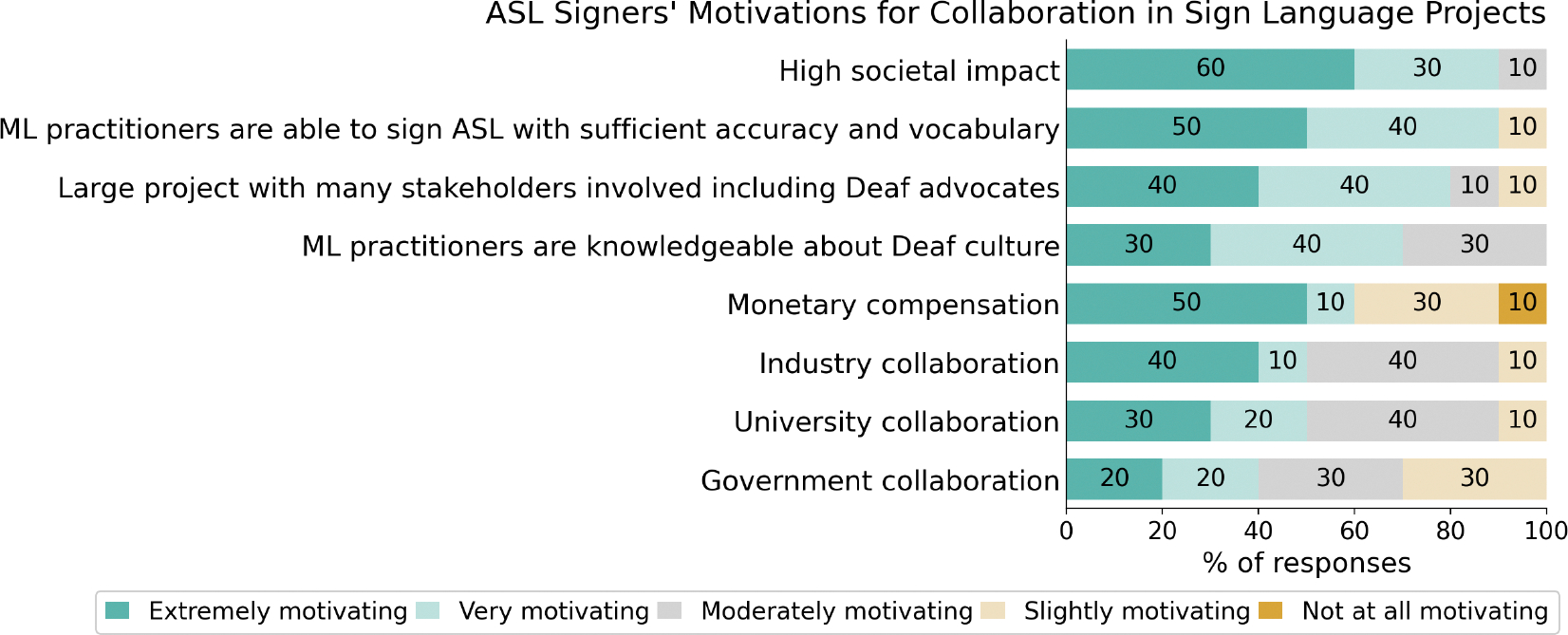
A breakdown of responses from ASL signers on the motivating factors for cross-cultural collaboration on sign language-related projects, each rated on a 5-point Likert scale (from ‘extremely motivating’ to ‘not at all motivating’).

Now, to understand **ASL signers’ motivations**, we specifically framed the questions around factors that could motivate them to collaborate on sign language projects. As shown in Fig. 3, motivations for collaboration included high societal impact (90%), concern about ML practitioners’ ASL proficiency (90%), and involvement of Deaf advocates (80%). While perspectives on compensation varied, 60% rated it as a strong motivator, consistent with prior findings on its importance for disabled contributors [61]. A similar trend was observed in our survey for collaboration entities, where the government was rated as less motivating than industry or universities, also consistent with prior findings indicating preference to support industry or universities in efforts to advance sign language AI [14].

In terms of **ASL signers’ concerns** collaborating with hearing people, communication-related issues were identified as the biggest barrier. This was often due to limited access to interpretation services, with one ASL signer noting *“No budget to pay”*, and inconsistent interpretation quality, leading them to opt for written text instead, though at the cost of losing *“the impromptu of it all.”* Considering these concerns, ASL signers expressed a desire for new (AI-infused) technologies for accurate real-time captioning and ASL-to-English translation. The existing barriers also reflected ASL signers’ preferred collaboration methods, which included video with text chat (50%) as a middle ground for live discussion paired with text. They also highlighted the importance of hearing collaborators’ awareness of Deaf culture (20%), with one ASL signer stressing the need to *“understand the Deaf perspective and the complexity of ASL.”*

### 4.2 Deaf Awareness

We assessed ML practitioners’ awareness of Deaf culture through true/false questions about terminology, identity, people, and language (ASL). Table 2 provides the questions along with a detailed breakdown of the percentage of correct responses. Overall, ML practitioners demonstrated varying levels of awareness:

#### Terminology:

Only some (31%) answered all questions correctly. Those who answered incorrectly included researchers in academia and industry, PhD students, and faculty members, with about half (46%) reporting experience in sign language processing. A notable misconception was considering ‘hearing impaired’ to be appropriate terminology, which is typically rejected by members of the Deaf community [35] due to negative emphasis on deficiency.

#### Identity:

The majority (69%) answered all questions related to Deaf identity correctly, indicating a stronger understanding compared to other sections.

#### People:

A minority (26%) answered all questions about people correctly, with the low rate largely due to incorrect responses (60%) to ‘Most deaf people who sign are bilingual’. Of those who answered incorrectly, nearly half (43%) had experience in sign language processing, ranging from months to more than 10 years. This may reflect the misconception that the Deaf community is not a culturally and linguistically distinct group [78].

#### Language:

About half of ML practitioners (54%) answered all ASL-related questions correctly. Misconceptions were observed but differed between those with and without experience in sign language processing. Those with no experience often mistook ASL for a direct representation of English or mime-like gestures; those with experience deemed ASL to be a universal language.

**Figure 4:**
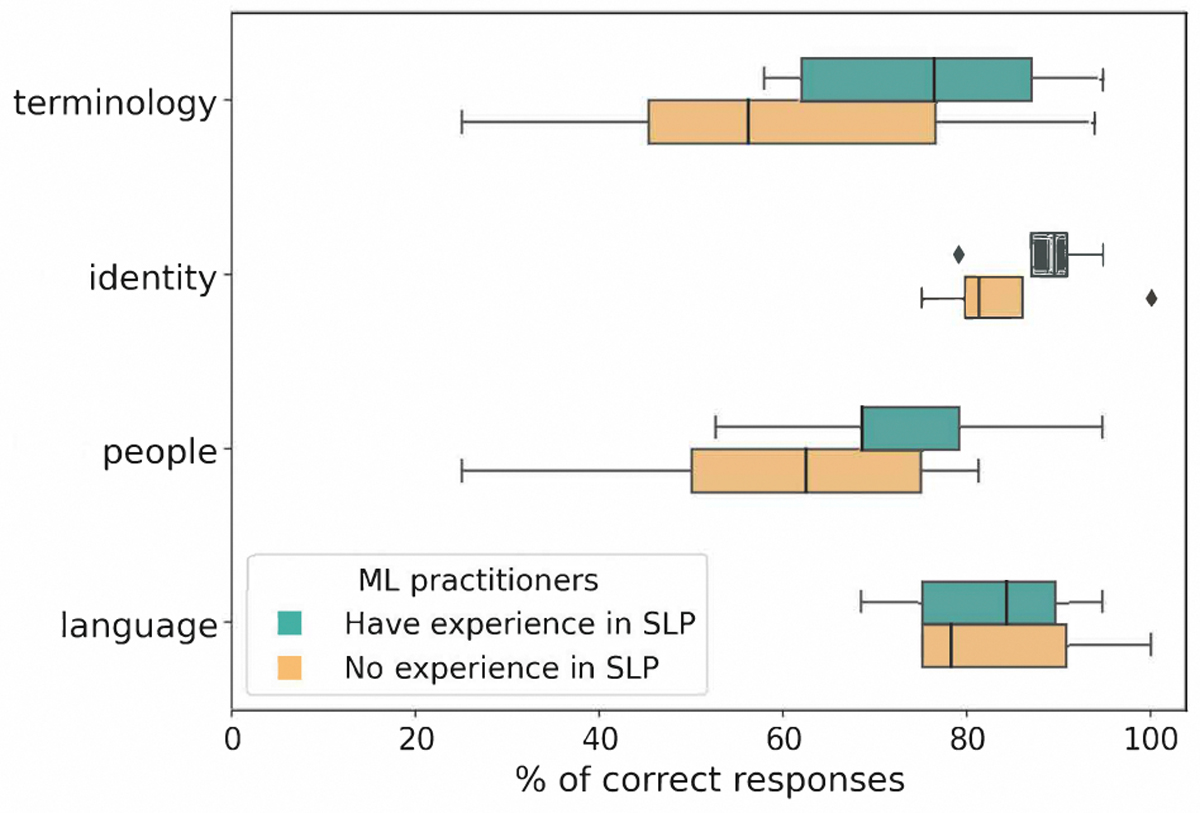
Box plots showing the distribution of percentage of correct answers to Deaf awareness questions by topic for ML practitioners with and without sign language processing (SLP) experience. Medians (black line) are consistently higher for those with SLP experience.

The overall trends in ML practitioners’ responses to Deaf awareness questions reveal misconceptions even among those with experience in sign language processing. While practitioners with such experience generally had higher median correct response rates across all sections (Fig. 4), statistical analysis (Mann-Whitney U test) din not reveal significant differences between the two groups: terminology (*U* =25, *p*=0.30), identity (*U* =10, *p*=0.66), people (*U* =17, *p*=0.40), and language (*U* =16, *p*=0.81).

### 4.3 Expectations in Sign Language Processing

**Figure 5:**
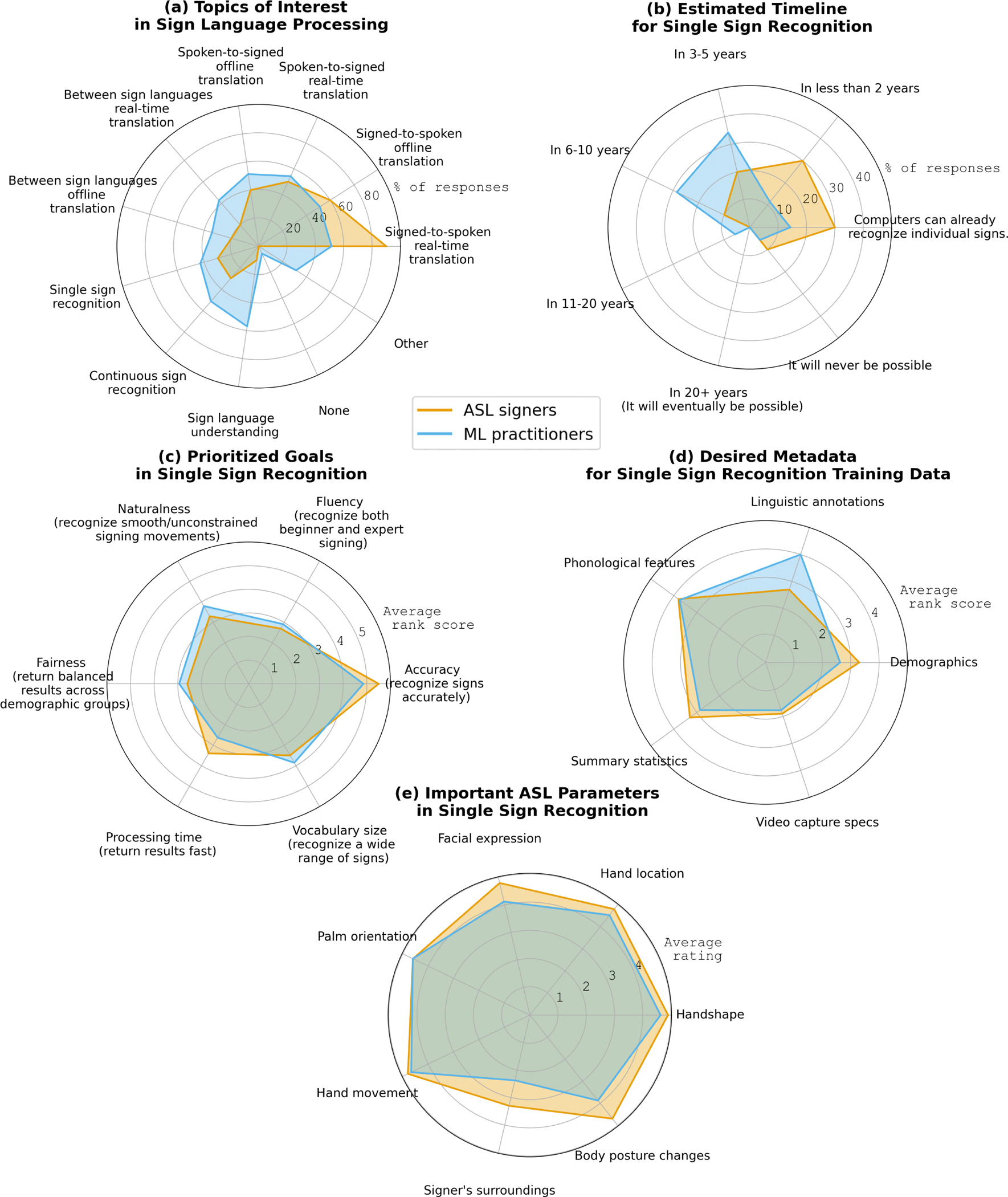
Comparison of ML practitioners and ASL signers’ responses in radial charts showing: (a) percentage of responses for project topic selection, (b) timeline estimation, (c) ranking of goals in sign language modeling, (d) ranking of important metadata for training data, and (e) ratings of ASL parameters.

We explored potential mismatches between ML practitioners and ASL signers in their perspectives on sign language processing tasks. In terms of **topics** (Fig. 5a), ASL signers showed clear preferences, with most (90%) prioritizing real-time translation from sign language to spoken/written language (*e.g.*, ASL to English). Half (50%) selected real-time translation in the opposite direction (*e.g.*, English to ASL). Looking closer, there is a clear asymmetry between recognition and generation tasks, which may reflect uneven technological progress related to the Deaf community. Recent advances in real-time captioning have improved access to spoken language, whereas sign language generation often relies on “prematurely released, unintelligible avatars” [50]. Signed-to-spoken translation—expressing oneself in sign language with real-time access for non-signers—still lags behind, making it a critical area for future work.

Among ML practitioners, the most commonly selected topic was sign language understanding including ASL grammar and lexicons (57%), a topic that, in contrast, was the least selected by ASL signers (10%). Preferences among ML practitioners were more diffuse; nearly half (51%) also selected either real-time or offline translation from sign language to spoken/written language. The least selected topic in this group was offline translation between sign languages (34%). They also expressed ’other’ interest toward applications, such as sign language learning for families and children and automating parts of linguistic research (*e.g.*, data annotation).

Next, we compared the groups’ perspectives on various requirements for single sign recognition development, as shown in Fig. 5b) timeline, c) goals, d) metadata, and e) training of ASL parameters. We focused on single sign recognition, as it can provide a baseline framework for more complex tasks, such as recognizing signs in continuous sequences. Overall, both groups showed similar expectations, though certain requirements reflect some cultural and disciplinary differences.

In **timeline estimation** for single sign recognition technology to match the proficiency of an average human signer, some ASL signers (30%) responded that computers have already reached this level or will do so within two years. In contrast, ML practitioners generally estimated a longer timeline, mostly selecting 3–5 (34%) or 6–10 years (29%). A small percentage in both groups (10% ASL, 6% ML) responded that this performance level will never be possible.

Regarding **goals**, both groups ranked accuracy as the top priority, with vocabulary size second. Few nuanced differences emerged in their ranking of speed—ASL signers placed it third, whereas ML practitioners ranked it lowest. Instead, ML practitioners prioritized naturalness third, which ASL signers ranked fourth, suggesting differing practical and technical perspectives. Regarding **metadata**, both groups valued phonological features, but ML practitioners ranked linguistic annotations higher, possibly reflecting greater familiarity with machine learning workflows.

Finally, in **ASL parameters**, ASL signers rated body posture changes highly (4.7), while ML practitioners rated them lower (3.9), indicating potential gaps in understanding sign language nuances. Both groups agreed that signers’ surroundings were less critical for recognition, potentially due to the limited state of sign language technology which is known to impact near-term priorities [13].

## 5 Co-design Findings

In total, we identified 10 main themes and 16 sub-themes of questions contributed by 8 ASL experts (ASL-P1 to ASL-P8) and 8 ML experts (ML-P1 to ML-P8) who participated in our co-design study. Appendix B details self-reported participant information, including expert type, ASL proficiency, and ML experience. Table 3 shows the percentage of contributors per theme, highlighting distinct focus areas shaping each group’s questions, as well as shared emphases that reveal overlapping perspectives. Broadly, ASL experts often emphasized project motivations, while ML mostly experts focused on objectives such as clarifying target users and use cases. Both groups also contributed questions on shared concerns, such as deadlines. For a comprehensive view, Appendix C presents a sub-theme breakdown with overlap percentages, highlighting areas where distinct viewpoints emerged and where further dialogue may be beneficial.

### 5.1 Motivation

#### Emphasis by ASL experts.

Participants, mainly ASL experts, posed questions that articulated the collective rationale for undertaking the project, often framed as *“Why are we doing this (project)?”* (ASL-P2, ASL-P3, ASL-P6, ASL-P8). This aligns with reflective practices described in frameworks for responsible and inclusive technology development [26], which encourage early discussions about stakeholder priorities across “different spheres” and potential tensions or tradeoffs in prioritizing one initiative over another.

#### Emphasis by both.

Building on the core question of project motivation, both ASL and ML experts reflected on its significance relative to alternatives and current needs with questions such as *“Why focus on this (project) other than something else?”* (ASL-P5) and *“Why is this (project) a priority now?”* (ML-P2).

### 5.2 Objectives

#### Emphasis by ML experts.

ML experts focused on narrowing project tasks, asking questions like *“What is the task we want to address? (Captioning, detection, translation to audible?)”* (ML-P5) and *“What are we doing? Text to sign or sign to text?”* (ML-P6). They also considered specific contexts for technology application, asking questions like *“Noisy or social settings?”* (ML-P4), to ground the project in real-world use cases. Interest in use-case discussions was a recurring theme among ML experts but appeared less prominently among ASL experts. This gap between the two groups could be explained by a concern expressed by one ML expert: *I think sign language projects would benefit from having a clear and well-defined use case that can only come from DHH experiences. One of the biggest problems in sign language processing at the moment is precisely that almost none of us have that specific perspective”* (ML-P6).

#### Emphasis by both.

Both ASL and ML experts questioned the project’s purpose by focusing on its target users and the diversity within the Deaf community. Questions like *“The DHH community is a spectrum. Who do you target when working on projects?”* (ASL-P5) emphasize the need for clarity in addressing the variations in sign language execution across factors such as ethnicity, region, age, gender, education, language proficiency, and hearing status [13]. Similarly, questions such as *“Where the project should be focused? One institution, one local community, country-wide?”* (ML-P3) prompt the appropriate scale and scope. While defining these specifics, participants also considered the project’s long-term impact, asking *“Where we end up in the future?”* (ASL-P1) and *“How will we build on this in the longer term?”* (ML-P7). These questions prompt stakeholders to consider how the project can scale and adapt to Deaf community’s evolving needs.

### 5.3 Gaps & Opportunities

#### Emphasis by ML experts.

ML experts critically questioned assumptions underlying the project, such as *“Why do we need AI for this?”* (ML-P6) and *“Why is tech solution the preferred way? Any alternatives?”* (ML-P4). These questions encourage a broader consideration of sociocultural contexts to prevent issues like cultural appropriation [79], where community needs may be overlooked in favor of technological innovation.

#### Emphasis by both.

Both ASL and ML experts raised questions to understand the current state of research and identify gaps the project could address. For example, *“What else is in the field right now? What’s been done? What type of data exists?”* (ASL-P3) and *“What kinds of projects have you seen that are similar?”* (ML-P3) guide discussions to situate the project within existing knowledge and clarify the target problem space.

### 5.4 Methodology

#### Emphasis by ASL experts.

ASL experts emphasized methodological scrutiny, raising questions about the rationale behind specific approaches: *“Why are we doing it this way?”* (ASL-P8). Deployment was a critical theme, with ASL experts highlighting concerns about user interaction and adoption. For example, an ASL expert questioned the learning curve with *“Why would the user want to re-learn new technology to do the same things?”* (ASL-P4). This highlights the potential resistance to solutions that require users to learn an entirely new interface or process, especially if it doesn’t integrate into their daily routines or improve upon current practices [48].

#### Emphasis by both.

Both focused on development, especially technical needs such as minimizing latency *e.g.*, *“How can we make all this happen in real-time or with a maximum of 1–2 seconds delay?”* (ASL-P2). Sensory modalities also drew interest. For example: *“What should the capture system look like?”* (ASL-P2) addresses input, while *“Will it have a human-like interface that can interact with an individual?”* (ML-P8) addresses output.

Platform considerations surfaced in questions such as *“Are we going to build something visible on iOS or web?”* (ML-P8). An ASL expert who had a similar question added a new perspective with *“Where will this be built on? Web? iOS? Android? Where will it be accessible?”* (ASL-P8), emphasizing availability on widely used platforms to enhance accessibility and adoption [72].

Data-related discussions revealed a shared focus among ASL and ML experts. They raised questions about data sources, such as *“Who should we recruit as references for the ASL signs?”* (ASL-P6) and *“How will the individuals be gathered?”* (ML-P8). These questions build upon existing ethical frameworks (*e.g.*, Datasheets for Datasets [33]) by highlighting the technical and strategic challenges of sourcing data directly from individuals. Discussions also addressed data diversity, such as *“How diverse should the data be? TV interpreters with the same outfit and backgrounds would suffice?”* (ML-P3). Answering such questions requires a thoughtful, ethical approach that balances technical needs with inclusivity and fairness, aligning with Responsible AI values [66].

Even when datasets are designed with representativeness in mind, they can still underrepresent certain groups due to the inherent variability in language proficiency among signers [12]. This concern is reflected in further questions on data quality and validation, such as *“Who will be assessing the data?”* (ASL-P7) and *“How do we create better datasets, and what tests are required before ML can really advance in this area?”* (ML-P4). Both groups shared awareness of the complexities in the field, particularly the ethical considerations and logistical challenges of data collection.

### 5.5 Scope & Outputs

#### Emphasis by ML experts.

ML experts raised key questions about defining the project’s scope and constraints, especially factors affecting implementation. Examples include: *“What other (technical, business) constraints, if any, do we need to be aware of?”* (ML-P2) and *“What are we going to be including, hands? Posture? What else? What are the different ways? What is all the information that we will and won’t be using?”* (ML-P2). They also questioned the project’s nature – *“Is this a research or commercial project?”* (ML-P6) – and considered end goals, asking if the focus should be on a *“Research portfolio, product development, open sourcing?”* (ML-P5).

#### Emphasis by ASL experts.

While scope-related questions overlapped, ASL experts took a broader, more conceptual perspective than ML experts. For example, one asked, *“Is it the people? Is it the function? Is it the exposure?”* (ASL-P3), contrasting with the ML experts’ technical, constraint-driven focus. Their perspective underscores the need for sign language technologies to address real-world constraints [13]. As one noted, *“I worry that solutions and products that are created for signed language technology will not spread ’out’ of the signed language technology field”* (ASL-P4), highlighting that constraints include community priorities and long-term needs, not just technical challenges.

### 5.6 Validation

#### Emphasis by ASL experts.

While both groups addressed model and system validation, ASL experts emphasized user experience and ethical responsibility. They prioritized structured testing phases with questions framed as ‘when’. Early evaluations involved the target community *e.g.*, *“When do we anticipate testing our technology with skilled sign language users?”* (ASL-P3). For later stages, they asked about readiness for deployment*e.g.*, *“When do we want to do alpha and beta testing?”* (ASL-P8) and *“When would the machine be mature for public use?”* (ASL-P7). Concerns for reliability emerged in questions like *“How often may we see slight mishaps?”* (ASL-P1). They also considered long-term oversight, asking *“Who do you think should regulate and track this machine in the long term?”* (ASL-P7).

#### Emphasis by ML experts.

In contrast, ML experts were more concerned with validation methodologies and identifying appropriate testing environments, often framed as ‘where’ questions such as *“Where are we going to deploy this project? Is it going to be in public? Or do we want a controlled environment to first test?”* (ML-P8) and *“Where do we want to deploy the project? (Use case context)”* (ML-P5). Unlike ASL experts, no question was made about monitoring and regulation, suggesting immediate deployment contexts as reflected in a question *“Where should we test/deploy the project?”* (ML-P7).

### 5.7 Timeline

#### Emphasis by ASL experts.

Their questions reflected an exploratory, collaborative approach to project planning. They asked, *“How shall we go about this project? How many ways can we think of to solve this problem?”* (ASL-P8) and *“How many possible ways/perceives to solve the project?”* (ASL-P1) seeking diverse approaches for effective solutions. *“Should we split up the project into tasks (e.g., one focusing on sign to text and the other text to sign)?” (ASL-P6)* further showed a holistic view of project structure and task division.

#### Emphasis by ML experts.

In contrast, ML experts focused on identifying key priorities early, asking *“What is the most important thing we need to get right?”* (ML-P2) and *“What are the key elements needed to focus on?”* (ML-P4). They also asked about deliverables, such as *“What is the first deliverable?’*’ (ML-P1) and *“When are we going to be able to complete the data collection and make the deliverables?”* (ML-P8). These questions contrasted with the exploratory focus of ASL experts, reflecting differing priorities that some ML experts saw as potential collaboration challenges. As one noted, *“Maybe dealing with too big expectations and not having intermediate, smaller and more feasible goals in the middle”* (ML-P3).

#### Emphasis by both.

Both groups emphasized defining milestones *e.g.*,*“When are our milestones? Target deadlines?”* (ASL-P5) and *“When are we expecting to have initial results by?”* (ML-P7). hey also stressed breaking large goals into manageable timeframes, as in *“What do we want to accomplish in the next four weeks?”* (ASL-P7) and *“When do we need an MVP by?”* (ML-P2). However, ML experts tended to focus on output-driven milestones with testable outcomes, while ASL experts framed them more broadly.

### 5.8 Stakeholder Analysis

#### Emphasis by ASL experts.

ASL experts prioritized Deaf community involvement and the project’s long-term impact. Questions like *“Where do Deaf people see AI going?’*’ (ASL-P2) and *“What would the Deaf community’s response be?”* (ASL-P8), reflect this focus. They also balanced internal collaboration with external community engagement while striving for inclusivity and cultural sensitivity, asking *“Who will be our mentors/supervisors?”* (ASL-P1) or *“Who is involved (to make sure we have a full team)? More representatives like international community and varied background?”* (ASL-P7).

#### Emphasis by ML experts.

While both groups raised questions on stakeholder engagement and team roles, ML experts focused on practical concerns such as data collection and internal operations *e.g.*, *“Who is going to collect the data?”* (ML-P8) and *“Who are our institutional contacts? (IRB, computer resources, vendors)”* (ML-P5). Focusing more on technical and logistical aspects of stakeholder involvement, ML experts prioritized understanding their motivations and interests, such as *“Why do we expect people to be interested in contributing to this project?”* (ML-P7). Questions like *“Where is the highest pain point for the community?”* (ML-P4) indicated an effort to identify areas of significant concern, but it could also suggest a problem-solving focus that risks overlooking the broader context of the community’s experiences and perspectives.

### 5.9 Benefits & Ethics

#### Emphasis by ASL experts.

ASL experts raised explicit ethical concerns asking, *“What are some of the ethical concerns to contend with?”* (ASL-P2). They placed a greater emphasis on balancing benefits, risks, and costs – *“What benefits and risks would this project bring?”* (ASL-P6) – and questioned financial accessibility: *“Who’s gonna cover those costs? Will the government cover those costs? Will deaf users have to pay for that cost? Is that accessible?”* (ASL-P8).

#### Emphasis by both.

While most ML experts’ questions lacked an ethical dimension, one – along with ASL experts – stressed the importance of positive community impact: *“Why is the task beneficial to the DHH community?”* (ASL-P5) and *“Why is this an important project for the mixed hearing ability community? Will this help anyone at all?”* (ML-P8). This shared focus invites discussion on practical and social significance, though ASL experts offered a more critical lens. As one noted, *“This could be a form of genocide. I only hope this would benefit us more than it would destroy us. Currently, our community celebrates the diverse [forms] of signing and characteristics. With human interpreters, we already have to code switch to ensure the L2 would understand. So, now with AI, what would that look like?”* (ASL-P7).

### 5.10 Logistics - Resources & Feasibility

#### Emphasis by ASL experts.

ASL experts reflected on both available resources and future needs: *“What type of resources do we have access to, currently? And then what will we need?”* (ASL-P3). They were also concerned with the technical infrastructure and platform support, asking *“What kind of platform are we going to be using? What might we need to build in order to support this project?”* (ASL-P2). The same participant considered ways to stretch resources for long-term sustainability: *“How can we make cuts when possible, to use the budget as long as possible?”* (ASL-P2).

#### Emphasis by ML experts.

ML experts also raised resource concerns, asking, *“Who is funding the project?”* (ML-P1), but focused more on immediate needs like data and evaluation: *“What are our resources? (Budget, data landscape, human evaluation?)”* (ML-P5). They linked resource availability to project scope, noting it can *“dictate the resources and budget”* (ML-P2).

### 5.11 Logistics - Collaboration

#### Emphasis by ML experts.

ML experts often asked structured, technical questions about collaboration management, such as *“How do we manage development? Git repos? Data storage, notes?”* (ML-P5). They focused on practical tools, communication platforms, and regular progress tracking *e.g.*, *“When is our next check-in?”* (ML-P2).

#### Emphasis by both.

Both groups raised questions about facilitating collaboration, including logistics for meetings *e.g.*, *“Where will we be able to do meetings?”* (ASL-P1) and *“Where are we going to be located/do we need a physical space for this?”* (ML-P6). Questions included effective communication methods *e.g.*, *“How should we facilitate collaboration?”* (ASL-P5) and *“What is the best way to communicate? What tools?”* (ML-P1). Unlike ML experts, ASL experts did not explicitly mention scheduled check-ins, suggesting they may view this collaboration as a more fluid process. One ASL expert reflected on its long-term trajectory: *“Where do we see the future of this collaboration unfolding?”* (ASL-P5).

## 6 Discussion

While addressing requirements from the outset to provide directions [52] is a success factor for projects, this is particularly challenging in interdisciplinary collaborations where efforts must be aligned under differing incentives and experiences among collaborators [18]. In this section, we discuss further considerations for collaboration and ethical practices, limitations of our work, and potential future directions to have meaningful involvement of the Deaf community in AI projects for sign language.

### 6.1 Implications

#### 6.1.1 Challenges and Opportunities for Collaboration.

Our work reveals both opportunities and challenges for collaborative practices in this space. While survey responses revealed a shared motivation to achieve real-world impact, each group navigated distinct tradeoffs (Sec. 4.1). For ML practitioners, the domain complexities of sign language processing and resource limitations (*e.g.*, datasets and benchmarks) pose significant barriers. Their work also tends to follow a top-down approach, shaped by lab priorities or funding constraints. These factors may influence their intermediary goals, prioritizing technical and linguistic needs over the broader cultural and practical context of sign language use. Additionally, their underlying motivation to apply or advance machine learning in this interdisciplinary domain can reinforce a technically driven approach. Indeed, our survey showed that many ML practitioners with experience in sign language processing lacked fluency in sign language and held misconceptions about Deaf culture (Sec. 4.2).

Drawing on insights from our survey, which further revealed gaps and differing expectations between ML practitioners and Deaf ASL signers (Sec. 4.3), our co-design sessions explored opportunities to support cross-disciplinary and cross-cultural collaboration. The guiding questions generated by ASL and ML experts surfaced shared priorities and areas needing further dialogue. These discussions may serve as meaningful entry points into complex problem spaces [18] and help future teams critically engage with stakeholder priorities [26], such as in defining project significance and addressing challenges like data representativeness. To support opportunities for future teams to align their efforts, we share the guiding questions grouped by (sub)themes^4^.

Despite aligned efforts, mismatches still emerge in how questions are framed around problem formulation and ideation. For example, both groups addressed project objectives, particularly concerning target users and diversity within the Deaf community. However, their approaches differed. ML experts placed a heavy emphasis on defining practical use cases. Yet, use-case discussions appeared less prominently among ASL experts.

Within the shared theme of ‘Scope & Outputs,’ ASL experts emphasized broader societal impact and long-term influence on the Deaf community. These considerations went beyond the technical feasibility and constraints that were more often raised by ML experts. For ASL experts, social and cultural implications, also surfaced in areas like ‘Timeline’ and ‘Logistics’. In contrast, ML experts took a more task-oriented inquiry approach, with a stronger emphasis on technical feasibility and task definition similarly to their questions around ‘Objectives.’ These different priorities may reflect unconscious assumptions held by Deaf experts and ‘unknown unknowns’ for ML experts, highlighting the need for intentional scaffolding in cross-disciplinary and cross-cultural teams [51, 82].

These tensions call for more structured reflexivity in how stakeholder roles and decision-making power are distributed. The importance of Deaf leadership has been extensively discussed in the literature [12, 37, 60]. The divergence we observed in our study can be another indicator to reassess collaboration norms involving Deaf communities. Desai *et al.* [24] urge hearing researchers to shift from dictating the agenda to supporting Deaf-led efforts while cautioning against tokenism. Our study aligns with this call but surfaces the importance of reciprocal learning in future collaboration. We draw on ASL-P3’s perspective, which envisions: *“Having collaborators who have emphasis on ASL take the time about educating themselves with ML, and ML/AI collaborators take the time to understand ASL/Deaf culture.”* Instead of fully transferring accountability to Deaf researchers, we advocate for transparency and shared understanding among team members throughout the project lifecycle [52]. Insights from our studies, especially the distinct emphases identified across communities, can help stakeholders anticipate misalignments and structure collaboration more intentionally.

#### 6.1.2 Ethical Considerations.

One critical finding from our co-design sessions is the relative absence of an ethical lens in the questions posed by ML experts. ASL experts emphasized the potential risks of AI on the Deaf community. Ethical concerns only surfaced around data-related issues for ML experts, echoing the growing discourse on AI fairness and representation within and beyond accessibility [11, 29, 39, 55]. ASL experts considered ethical dimensions broadly, as seen in their discussions of ‘Stakeholder Analysis.’ Their questions often balanced internal team reflections with outward-facing concerns. This holistic framing stands in contrast to ML experts’ focus on optimizing task distribution within the team and managing practical considerations. While certainly relevant for projects, these technical concerns can risk sidelining community priorities if they dominate early-stage planning. Previous literature has argued that community engagement often occurs too late—after significant development work has been done [30, 31].

While ML experts still raised questions about community benefits and impact, these were often framed through the lens of project motivation rather than accountability. This may be reflective of critiques by Whittaker *et al.* [83], noting that grant proposals often reference impact but rarely follow through on whether or how that impact is realized. A related concern is the recurring narrative that centers on claims in the assistive context, such as *“helping disabled people”* [83] or *“fixing”* [53] to create impact statements. This perspective was echoed by ASL-P5 during our co-design study: *“Looking forward to more and more ML experts getting a better understanding of ASL and the community to ensure we do not get more grant money towards well intended but unbeneficial efforts.”*

To address this, we advocate for discussions inspired by ASL experts’ questions. Singleton *et al.* [73] argue that it is crucial for researchers to give back to the Deaf community, aligning with broader efforts to *decolonize* research [8]. This perspective reinforces the call to shift from ‘inclusion’ to ‘agency and control’ given the proprietary nature and centralization of power inherent in AI systems [83]. This sentiment is captured in ASL-P7’s comment: *“This could be a form of genocide…So, now with AI, what would that look like?”* As language technologies risk centralizing or flattening linguistic variation, these concerns around language erasure and cultural threat must become central to the design process.

### 6.2 Limitations and Future Work

Several limitations shape the scope and interpretation of our findings. First, our study does not capture the full arc of long-term collaboration, particularly how relationships and power dynamics evolve beyond the problem formulation and ideation stage. We also did not explore the initial team composition process or how to best select team members. Our participants were brought into an already structured environment with Deaf and hearing facilitators and established turn-taking. This may have biased our findings toward more idealized collaboration experiences and does not reflect the broader sign language AI landscape, where hearing researchers often work without Deaf input or dominate the discussions.

Second, our relatively small sample size, with participants mostly from academic institutions, may limit the applicability of our insights to sectors like government-community or industry-community collaborations. While small sample are common in HCI, especially with underrepresented or expert communities [15], our participant mix reflects additional challenges. For example, in our survey, we had 10 ASL signers, 19 ML practitioners with sign language processing experience, and 16 without. This imbalance partly reflects our effort to address educational parity: although only about 23% of Deaf adults in the U.S. hold a bachelor’s degree or higher [10], all signers in our survey did, better aligning with the typically graduate-level education of ML professionals.

Ideally, we would have liked to recruit ASL experts with direct AI expertise. However, significant structural barriers in the training pipeline [24] make this virtually impossible; there are not many such individuals. Instead, we recruited highly informed, professionalized DHH ASL signers to reflect on these technologies’ impacts in their communities. Most held at least a bachelor’s degree, with 28% having completed graduate education–making this an unrepresentative sample of the broader Deaf community [17].

Third, while this is not unique to our research, sign languages are inherently diverse and tied to various cultural norms, making it challenging to generalize findings across different regions or linguistic groups [79]. To gain a more comprehensive understanding, further research should explore the various nuances of collaboration, including the challenges and opportunities that arise within government, industry, and community partnerships.

Last, the positionality of our analysis must be acknowledged. While our study team consisted of both Deaf and hearing researchers, the interpretation of the questions may reflect an insider perspective on technical norms that often shape AI research (*e.g.*, supporting the ML community in collaboration). This framing risks reinforcing dominant assumptions or overlooking deeper Deaf cultural nuances and epistemological standpoints. Rather than treating inclusion as a one-way effort, we need reciprocal approaches that support Deaf leadership and sustained roles for Deaf people in shaping both the research questions and the methods used to explore them. Future work should expand these discussions by involving a wider range of stakeholders, such as linguists, Deaf advocates, and Deaf professionals across sectors. These efforts are ongoing [20, 24], and are part of a larger body of work to facilitate interdisciplinary approaches and actionable guidelines that empower the Deaf community to shape the discussion in sign language AI.

## Figures and Tables

**Table 1:** Surveys for ML practitioners and ASL signers with overlapping themes of questions.

Question Themes	ML Practitioners	ASL Signers
Background	demographics; deaf identity and ASL proficiency	
	experience in ML and sign language processing; awareness of Deaf culture	experience in cross-cultural & cross-disciplinary collaboration; awareness of ML
Motivations / Challenges	ML and sign language processing projects	collaboration projects
Expectations in Sign Language Processing	topics: problems to work on; timeline: outlook on technology advancement; modeling: goals and tasks	

**Table 2:** ML practitioners’ responses to culturally appropriate terminology, identity, people, and language (specifically, ASL). Correct response rates for true/false items are shown as aggregated rates for all questions within each section, as well as individual question rates. Note: The order of true/false items in the survey differs from the order shown in this table.

*Select the term(s) or statement(s) that are true*	Correct Answer	Correct Response Rate

**Culturally appropriate terminology:**		**31%**
Deaf [with a capital “D”]	True	71%
deaf [with a lower case “d”]	True	51%
hard of hearing	True	54%
hearing impaired	False	51%
communication impaired	False	86%
mute	False	94%

**Identity:**		**69%**
“Deaf” generally refers to the group of people who share a language...	True	83%
“deaf” generally refers to the audiological condition...	True	80%
“Deaf” generally refers to the audiological condition...	False	86%
“deaf” generally refers to the group of people who share a language...	False	97%

**People:**		**26%**
Not all deaf people use sign language.	True	74%
More than 90 % of deaf children are born to hearing parents.	True	66%
Most deaf people who sign are bilingual.	True	40%
Most deaf people can read lips to communicate.	False	66%
Most deaf people would like their hearing to be cured.	False	86%

**Language:**		**54%**
ASL utilizes space and movements to convey meaning.	True	89%
ASL has regional accents and dialects.	True	74%
ASL is a universal language.	False	74%
ASL is a visual representation of spoken English.	False	77%
ASL relies on mime or picture-like gestures.	False	83%
ASL has the same grammar system as English.	False	94%

**Table 3:** An overview of unique and shared emphases of discussion by ASL experts and ML experts.

Themes & Contributing Experts	Emphasis by ASL Experts	Emphasis by ML Experts	Emphasis by Both
**Motivation** ASL: 6 (75%) ML: 3 (38%)	Clarified the collective rationale for undertaking the project	–	Prompted evaluations of project relevance and priorities
**Objectives** ASL: 6 (75%) ML: 7 (88%)	–	Focused on defining use cases	Focused on defining target users and scale
**Gaps & Opportunities** ASL: 1 (13%) ML: 3 (38%)	–	Examined assumptions & considered non-tech solutions	Explored research gaps
**Methodology** ASL: 6 (75%) ML: 6 (75%)	Focused on approach rationale & deployment concerns	–	Focused on technical requirements *e.g*. latency, platform accessibility, data sourcing & diversity
**Scope & Outputs** ASL: 3 (38%) ML: 5 (63%)	Broadened conceptual focus	Defined scope via constraints	–
**Validation** ASL: 4 (50%) ML: 4 (50%)	Focused on user experience and accountability	Focused on testing environments	–
**Timeline** ASL: 7 (88%) ML: 8 (100%)	Adopted an exploratory approach to project planning	Focused on identifying key priorities and deliverables	Focused on clear, manageable milestones, with questions about deadlines & outcomes
**Stakeholder Analysis** ASL: 6 (75%) ML: 6 (75%)	Prioritized inclusivity and the project’s long-term impact	Focused on internal operations	–
**Benefits & Ethics** ASL: 4 (50%) ML: 1 (13%)	Highlighted risks vs. benefits	–	Reflected on impact
**Logistics** ASL: 5 (63%) ML: 4 (50%)	Examined long-term resource needs	Focused on immediate needs and structured coordination	Raised questions about meeting logistics and tools

## Footnotes

1 gloss is a form of transliteration where written words in another language (*e.g.*, English) are used to provide semantic labels to signs (*e.g.*, ASL).

2 Liz Jackson calls the “Disability Dongle: A well intended elegant, yet useless solution to a problem we [disabled people] never knew we had” [40].

3 We use the term ’experts’ to refer to participants in the co-design sessions, recruited based on relevant expertise (see criteria in Sec. 3.2.1). This framing does not imply that they identify or describe themselves as experts in the field.

4 Available at https://iamlabumd.github.io/ask4asl

## A Survey Participant Characteristics

### Identity.

All ASL signers (N=10) identified as Deaf. Among ML practitioners (N=35), 2 participants identified as Deaf but primarily used sign languages other than ASL (German and Greek Sign Language), and 1 reported being deaf in one ear. The remaining 32 ML practitioners identified as hearing.

### ASL Proficiency.

Among ASL signers, not all rated their ASL proficiency at the highest level (Level 5, equivalent to a sophisticated native signer); 60% did so. The remaining 40% reported Level 3 or 4, indicating they could participate effectively in most conversations (Level 3) or use ASL fluently and accurately for professional needs (Level 4).

Among ML practitioners, most (88%) rated their ASL proficiency at Level 0, indicating they are unable to function in the language. Two ML practitioners self-rated their ASL proficiency at Level 3 and Level 4—one Deaf and the other hearing.

### Gender, Education, and Age.

Table 4 provides a breakdown of demographics by gender, highest degree earned, and age group. Likely influenced by our snowball sampling method, we see a skew toward higher education levels in both groups, with the majority (60%) of participants in the ASL group holding a Bachelor’s degree. Most (80%) in the ML group have a Master’s or Doctorate, often including academics such as university professors, PhD students, or researchers. Occupations reported in the ASL group included project managers, university staff, and teachers in ASL or math. With a higher number of working professionals in both groups, there is a skew toward certain age categories, primarily between 25 to 44 (ML: 82%, ASL: 70%).

**Table 4:** Demographics of ML practitioners (N=35) and ASL signers (N=10) by gender (women, men, and non-binary), highest degree earned for education, and age group.

Gender			Education			Age Group		

	ML	ASL		ML	ASL		ML	ASL
W	43%	30%	High school diploma	0%	0%	18–24	6%	10%
M	49%	60%	Bachelor’s degree	17%	60%	25–34	54%	30%
NB	8%	10%	Master’s degree	37%	30%	35–44	28%	40%
			Doctorate degree	43%	10%	45–54	9%	10%
			Prefer not to say	3%	0%	55–64	3%	10%
						65+	0%	0%

### Prior Experiences.

Among ML practitioners, the most common fields of experience were NLP (66%), deep learning (63%), and computer vision (40%). More than half (54%) also have had some degree of experience in sign language processing, with the majority of them (78%) currently working on related topics. However, their ASL proficiency did not align; the majority of self-ratings (74%) from those with experience in sign language processing were at Level 0, indicating they are unable to function in the language.

In terms of prior experiences among ASL signers, half (50%) reported having taken computer science courses, and the majority (70%) self-rated their familiarity with machine learning high, indicating to have either a broad understanding of its concepts or extensive knowledge of its applications. Most (80%) also reported having collaborated on projects with hearing people from different domains. The most common domains for these collaborations were technology and engineering (60%), followed by arts (30%), natural/medical science (30%), and education (10%).

## B Co-design Participant Characteristics

Table 5 presents self-reported demographic and professional information of ASL and ML experts who participated in paired co-design sessions.

### ASL Experts.

Participants (ASL-P1 to ASL-P8) all identified as Deaf and use ASL as their primary language, with 6 from academia and 2 from industry. The majority of participants self-rated their ASL proficiency at levels 4 and 5, indicating advanced to native ASL proficiency. All reported prior involvement in technology-related projects, mostly with minimal ML experience. 6 participants mentioned working on projects related to sign language, 3 on projects related to Deaf people but not sign language, 1 on projects related to other disability communities, and 2 on projects unrelated to accessibility or disability. In these projects, all but one participant collaborated with hearing people.

**Table 5:** Information about ASL and ML experts, including IDs for participant pairs in co-design sessions, age group, gender, education, occupation, ASL proficiency (based on the ASL Language Proficiency Interview scale [80], from level 0—unable to function—to level 5—sophisticated native signer), and years of ML experience. A dash (-) indicates that the participant did not consent to disclose.

Expert-PID	Age Group	Gender	Education	Occupation	ASL Proficiency	ML Experience

ASL-P1	18–24	man	high school	undergraduate student	level 4	0
ASL-P2	45–54	man	bachelor	IT security officer	level 5	less than 1 year
ASL-P3	18–24	woman	bachelor	PR manager; accessibility consultant	level 4	1–2 years
ASL-P4	25–34	woman	doctorate	postdoc researcher	level 4	4–6 years
ASL-P5	25–34	man	some college	research assistant	level 3	less than 1 year
ASL-P6	25–34	woman	bachelor	research assistant	level 5	less than 1 year
ASL-P7	25–34	woman	bachelor	translation & marketing coordinator	level 5	less than 1 year
ASL-P8	35–44	non-binary	bachelor	business consultant	level 5	0

ML-P1	45–54	woman	master	lecturer	level 1	2–4 years
ML-P2	-	-	-	-	level 0	10+ years
ML-P3	18–24	man	master	PhD student	level 1	2–4 years
ML-P4	55–64	man	master	product manager	level 0	4–6 years
ML-P5	25–34	man	master	PhD student	level 0	2–4 years
ML-P6	25–34	man	bachelor	PhD student	level 1	4–6 years
ML-P7	25–34	woman	master	PhD student	level 0	4–6 years
ML-P8	25–34	man	master	PhD student	level 0	4–6 years

### ML Experts.

Of the participants (ML-P1 to ML-P8), 6 were from academia, and 2 were from industry. Most academic participants were PhD students or researchers, though some also worked as ML engineers in industry. Most reported 2–6 years of machine learning experience, with one reporting over 10 years. As expected, ML experts had lower ASL proficiency (levels 0 or 1) but had some exposure to Deaf culture or sign language. Specifically, 3 had taken ASL or Deaf studies classes, 2 had friends or family members who are Deaf or had attended Deaf community events, and 3 had education or work experience in sign language linguistics. Despite their experience in technology-related work and some exposure to Deafness, only half had collaborated with Deaf people in the past.

## C Question Contributions

Table 6 lists the main themes and sub-themes of questions contributed by ASL and ML experts.

**Table 6:** Overview of ASL and ML group question contributions across themes and sub-themes.

Main Theme	Sub-Theme	Question Overlap %	Example Questions
**Motivation**		100%	“Why is this (project) a priority now?” (ML-P2)
**Objectives**	Target users Use cases* End goals	100% 38% 100%	“The DHH community is a spectrum. Who do you target when working on projects?” (ASL-P5) “What are we doing? Text to sign or sign to text?” (ML-P6) “What is the final outcome we want for whoever we are building for?” (ASL-P8)
**Gaps & Opportunities**	Prior work Questioning solutionism*	100% 0%	“What else is in the field right now? What’s been done? What type of data exists?” (ASL-P3) “Why is tech solution the preferred way? Any alternatives?” (ML-P4)
**Methodology**	Rationale Model development Deployment Data/data contributors	0% 100% 75% 100%	“Why are we doing it this way?” (ASL-P8) “How can we make all this happen in real-time or with a maximum of 1–2 seconds delay?” (ASL-P2) “Are we going to build something visible on iOS or web?” (ML-P8) “What data is collected for training any ML?” (ML-P4)
**Scope & Outputs** *		50%	“Where do you focus the project on? Is it the people? Is it the function? Is it the exposure?” (ASL-P3)
**Validation**		40%	“Who do you think should regulate and track this machine in long term?” (ASL-P7)
**Timeline**	Approach & priorities* Deadlines	25% 71%	“When are we going to be able to complete the data collection and make the deliverables?” (ML-P8) “When are we expecting to have initial results by?” (ML-P7)
**Stakeholder Analysis**	External stakeholders Internal researchers* Community engagement*	67% 43% 0%	“Who should our stakeholders be through this process?” (ASL-P5) “What roles does each person have?” (ML-P1) “What would the Deaf community’s response be?” (ASL-P8)
**Benefits & Ethics** *		50%	“What benefits and risks would this project bring?” (ASL-P6)
**Logistics**	Resources/feasibility* Collaboration	43% 60%	“What is the budget?” (ML-P2) “Where are we going to be located/do we need a physical space for this?” (ML-P6)

An asterisk (*) marks (sub)themes with 50% or less overlapping content, indicating differences in approach, focus, or framing between the two groups.
